# Chemiluminescence behavior of CdTe-hydrogen peroxide enhanced by sodium hypochlorite and sensitized sensing of estrogens

**DOI:** 10.1186/1556-276X-9-201

**Published:** 2014-05-01

**Authors:** Bo Ling, Jianhong Bi, Zongxin Pi, Huaze Dong, Ling Dong

**Affiliations:** 1 Department of Chemical and Chemical Engineering, Hefei Normal University, Hefei 230061, People's Republic of China

**Keywords:** CdTe, Sodium hypochlorite, Estrogens, Chemiluminescence

## Abstract

It has been found that sodium hypochlorite enhanced the chemiluminescence (CL) of the CdTe nanocrystal (NC)-hydrogen peroxide system and that estrogens inhibited these CL signals in alkaline solution. CL spectra were used to investigate the mechanism of the CL enhancement. On the basis of the inhibition, a flow-injection CL method has been established for determination of three natural estrogens.

## Background

Estrogens are necessary for ovarian differentiation during critical developmental windows in most vertebrates and promote the growth and differentiation of the adult female reproductive system [1]. Natural and synthetic estrogens have been characterized by the largest endocrine disrupting potential, as confirmed by both in vitro and in vivo studies [2]. The relation between estrogens and several human health problems has been previously reported, such as prostate and breast cancer, perturbation of human reproduction, and endocrine disruption on humans and wildlife [3]. Estrone, estradiol, and estriol are three main natural estrogenic hormones existing in the human body. In the past years, they had been used widely as some regulatory factors preventing the aging substance in women and remedies related to women diseases.

Estrogens have been detected with some analytical procedures, including high-performance liquid chromatography [4-9], UV derivative spectrophotometric method [10], gas chromatography (GC)-mass spectrometry (MS) analytical method [11], and capillary electrophoresis [12]. Semiconductor nanocrystals have been widely used as fluorescence biological probes [13], donors or acceptors of fluorescence resonance energy transfer [14], and in bioimaging [15]. The reduced and oxidized nanocrystals, generated at a certain electrochemical potential, can react through the annihilation process or react with some co-reactants to produce electrochemiluminescence (ECL) [16-20]. The chemiluminescence (CL) of CdTe nanocrystals (NCs) induced by direct chemical oxidation and its size-dependent and surfactant-sensitized effect in aqueous solution were investigated [21]. Since the low luminous efficiency of the direct chemical oxidation, CdTe NCs' chemiluminescence reaction was enhanced by the Tween 20, sulfite, and some metal ions [22-24].

In this work, we found that sodium hypochlorite could enhance the CL of the CdTe NCs-hydrogen peroxide system. The results indicated that the CL emission intensity of CdTe-hydrogen peroxide-sodium hypochlorite system could be inhibited by estrogens. Therefore, the development of a CL system for determination of estrone, estradiol, and estriol was established, and the mechanism was also discussed.

## Methods

### Reagents and solutions

Estrogens were purchased from Sigma (St. Louis, MO, USA) and used without further purification. Stock solutions of estrone, estradiol, and estriol were firstly dissolved using several drops of 0.01 mol/L NaOH solution and the working standard solution was diluted with water. Sodium hypochlorite (NaClO) and H_2_O_2_ were purchased from Beijing Chemical Reagents Company, Beijing, China. The stock solution (H_2_O_2_) was standardized by titration with a standard solution of KMnO_4_. All reagents were of analytical grade and the water used was doubly distilled.

### Apparatus

All CL measurements were performed on the IFFM-E mode flow-injection chemiluminescence (FI-CL) analysis system (Xi'an Remax Company, Xi'an, China). It has two peristaltic pumps and one injection system synchronized by a microprocessor. All the reactor coils were made of Teflon tubing. The flow cell was a glass tube (i.d. 0.5 mm) connected with a selected high sensitivity, and low-noise photomultiplier tube. Light measurement data (ICL) were transferred to a computer automatically. Data acquisition and treatment were used with REMAX software running under Windows XP. The photoluminescence spectra and UV-visible absorption spectra were performed on a model F-4500 spectrofluorometer (Hitachi, Tokyo, Japan) and a model UV-3010 spectrophotometer (Hitachi, Japan), respectively. The transmission electron microscopy (TEM) images of the nanoparticles were acquired on a JEM-2010 F microscope. The CL spectrum was detected and recorded by a BPCL-2-KIC Ultra-Weak Luminescence Analyzer (Institute of Biophysics, Chinese Academy of Sciences) and combined with a flow injection system.

### Procedure

**Figure 1:**
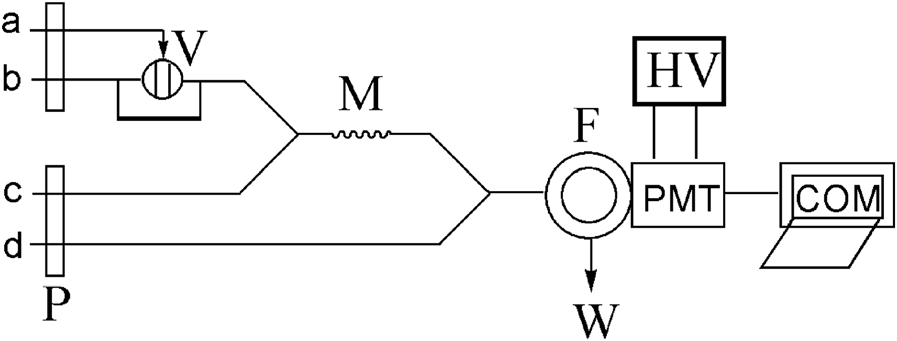
**NaOH (or sample solution) (a), CdTe solution (b), NaClO solution (c), and H**_**2**_**O**_**2 **_**solution (d).**

**Figure 2:**
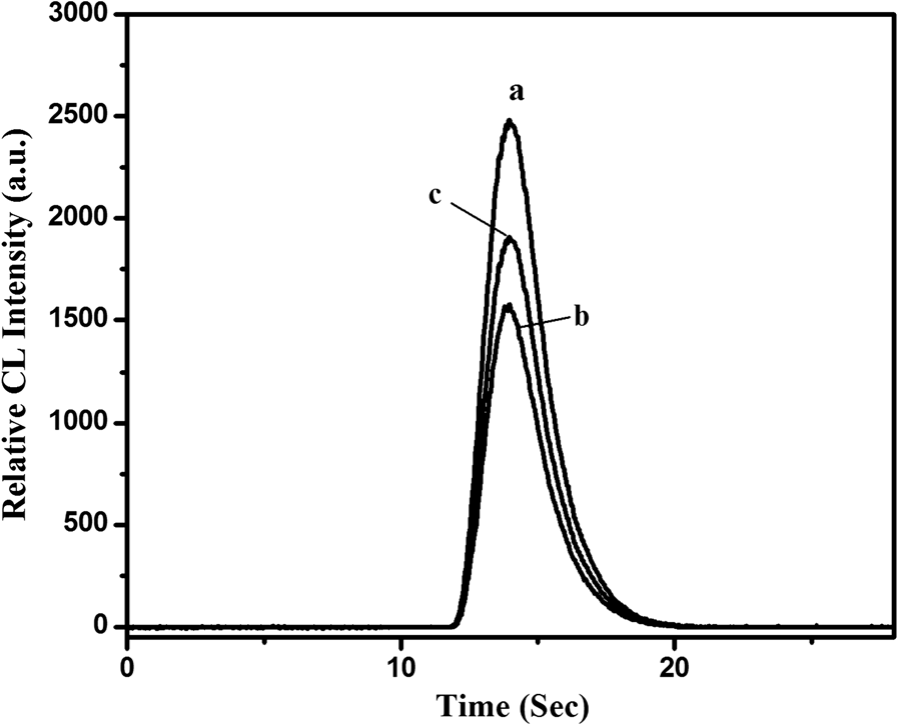
**CL kinetic curves of H**_**2**_**O**_**2**_**-CdTe NC CL reaction.**

A schematic diagram of the flow system was shown in Figure 1, in which four flow tubes were inserted into the NaOH (or sample) solution, CdTe NCs solution, H_2_O_2_ solution, and NaClO solution, respectively. One peristaltic pump (two channels) was used to carry NaOH (or sample) solution and CdTe NC solution, and another pump (two channels) was used to carry H_2_O_2_ solution and NaClO solution, respectively. The pumps were started with the flow rate of 2.5 mL/min for several minutes until a stable baseline CL curve was recorded. The CdTe-H_2_O_2_ system could emit weak CL in NaOH solution (Figure 2b). However, when NaClO solution of 1.27 × 10^-2^ mol/L was mixed with the CdTe_,_ and then injected into the stream, the CL signal was greatly enhanced (Figure 2a). Therefore, it could be assumed that NaClO strongly catalyzed the CdTe-H_2_O_2_ CL reaction. When estrogens were added to this CL system, the CL intensity decreased dramatically (Figure 2c).

## Results and discussion

### Synthesis of GSH-capped CdTe NCs

**Figure 3:**
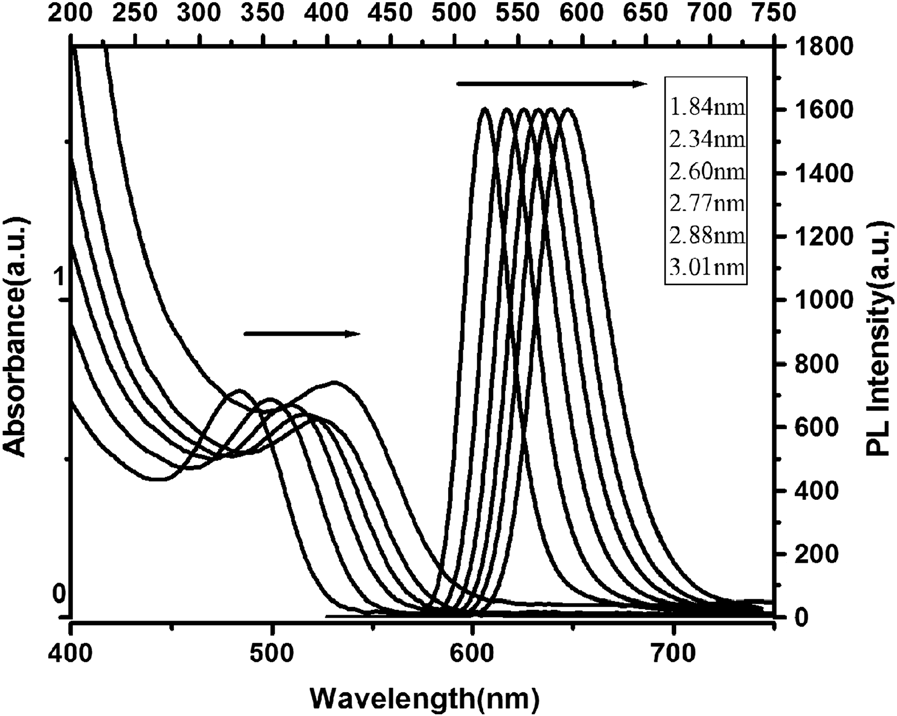
UV–Vis absorption and PL spectra of CdTe NC solution with different sizes of CdTe NCs.

A series of aqueous colloidal CdTe solution were prepared using the reaction between Cd^2+^ and NaHTe solution following the described method previously [21,25-27], and little modification was made. Cd^2+^ precursor solutions were prepared by mixing solution of CdCl_2_ and GSH (used as stabilizer), then adjusted to pH 8.0 with 1 M NaOH. The typical molar ratio of Cd^2+^/Te/GSH was 4:1:10 [28] in our experiments. This solution was placed in a three-necked flask, fitted and deaerated with high-purity nitrogen bubbling for 30 min. Under vigorous stirring, the prepared oxygen-free NaHTe solution was injected. The resulting mixture solution was heated to 90°C and refluxed at different times (2.5 to 9 h) to control the sizes of CdTe NCs [28]. Aliquots of the reaction solution were taken out at regular intervals for further UV absorption and fluorescence characterization (Figure 3).

### UV and PL characterizations of CdTe NCs

In Figure 3, the absorption and photoluminescence (PL) spectra of the different sizes of GSH-capped CdTe NCs were presented. All colloids obtained possess a well-resolved absorption maximum of the first electronic transition indicating a sufficiently narrow size distribution of the CdTe NCs. The absorption maximum and the PL peak shift to red wavelengths with increasing NC size as a consequence of quantum confinement. According to Peng's report [29], the particle size of CdTe NC was calculated using the following equation:

$$\begin{array}{ll} \;D & =\left(9.8127\times 10^{-7}\right)\lambda^{3}-\left(1.7147\times 10^{-3}\right)\lambda^{2}\\ & \;+\left(1.0064\right)\lambda -194.84 \end{array}$$

The sizes of the abovementioned CdTe NCs were around 1.84, 2.34, 2.60, 2.77, 2.88, and 3.01 nm, respectively, corresponding with the PL peaks of 524, 540, 554, 566, 575, and 589 nm (Figure 3).

### TEM characterization of CdTe NCs

**Figure 4:**
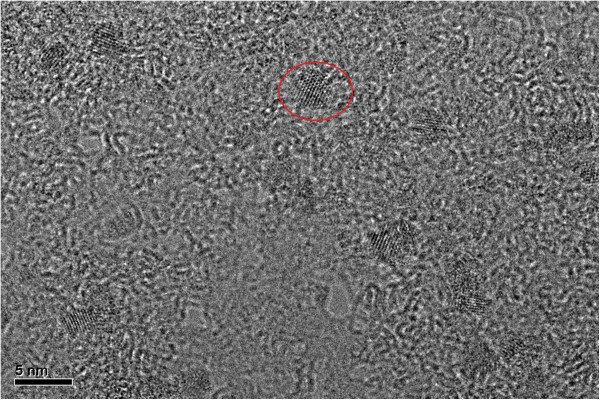
**TEM of CdTe, *λ***_**em**_ **= 554 nm.**

The CdTe NCs was also studied carefully by TEM (Figure 4). The morphology and size of CdTe QDs could be observed clearly, and the average size of studied CdTe NCs was about 2.60 nm. Considering that the value closing to 2.60 nm resulting from the empirical formula, it seems to be convenient to calculate the size of CdTe NCs.

### Effect of CdTe's size

**Figure 5:**
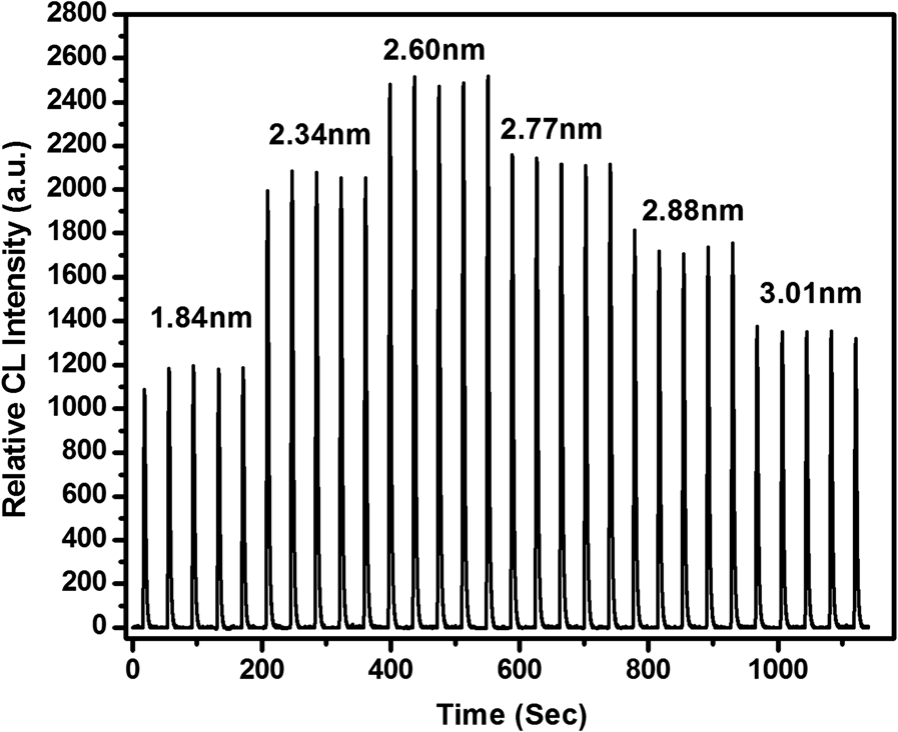
CL curves of CdTe NC solution with different sizes.

Size effect is a basic characteristic of semiconductor nanocrystals. A mass of researches have demonstrated that the optical properties of semiconductor nanocrystals are size-dependent [21,29-32], and so an experimental investigation of the size effect on CL response was conducted in the present work. Under the optimized conditions by the FIA-CL mode, the response of the abovementioned different-sized CdTe NCs to the CdTe NCs-H_2_O_2_-NaClO CL system was investigated as shown in Figure 5. The maximum CL intensity could be obtained when the CdTe diameter is 2.60 nm, which indicates that CL intensity of CdTe NCs has a size-dependent effect (Figure 5). The concentration of CdTe NCs, here, was fixed to 2.5 × 10^-4^ mol/L.

### Effect of CdTe NC concentration

**Figure 6:**
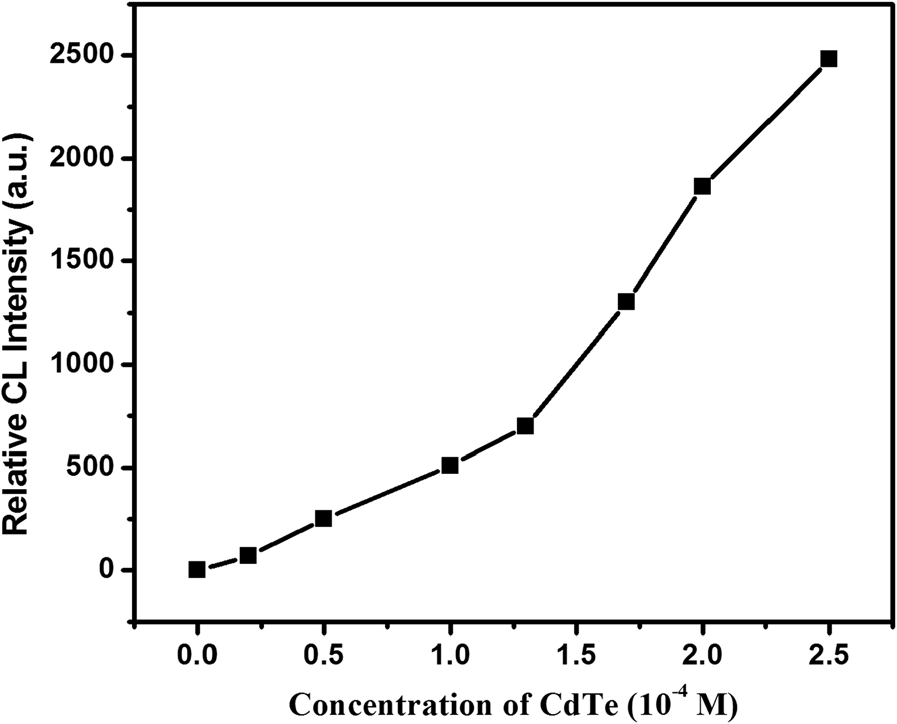
Effect of CdTe NC concentration.

The response of different concentrations of CdTe NCs to the present CL system was investigated under the optimal reaction conditions. It was found (Figure 6) that the CL intensity increased along with the increased concentrations of CdTe NCs in the range of 0 ~ 2.5 × 10^-4^ mol/L. The effect of CdTe NC concentration was studied (Figure 4). The CL intensity gradually increased as the CdTe NC concentration increased in the range of 0 ~ 2.5 × 10^-4^ mol/L CdTe (referring to Cd^2+^), which might be caused by a much higher concentration of CdTe NCs and generated more luminophor. In order to get a higher sensitivity, the concentration of 2.5 × 10^-4^ mol/L was recommended in this assay.

### Effect of hydrogen peroxide concentration

**Figure 7:**
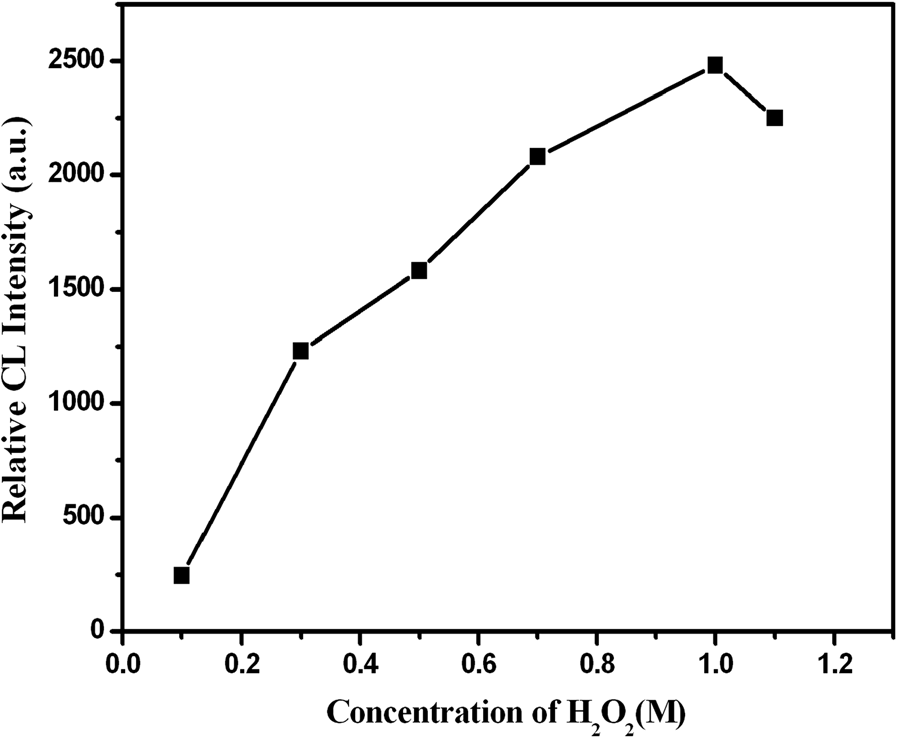
**Effect of H**_**2**_**O**_**2 **_**concentration.**

The concentration of hydrogen peroxide (H_2_O_2_) was optimized in the range of 0.1 ~ 1.1 mol/L in a FIA-CL mode described in the experimental section. As shown in Figure 7, the CL intensity continued to increase with the increase of H_2_O_2_ concentration up to 1.0 mol/L, then decreased. In order to get larger CL response signal and lower background signal, the concentrate of H_2_O_2_ 1.0 mol/L was used in the work.

### Effect of sodium hypochlorite concentration

**Figure 8:**
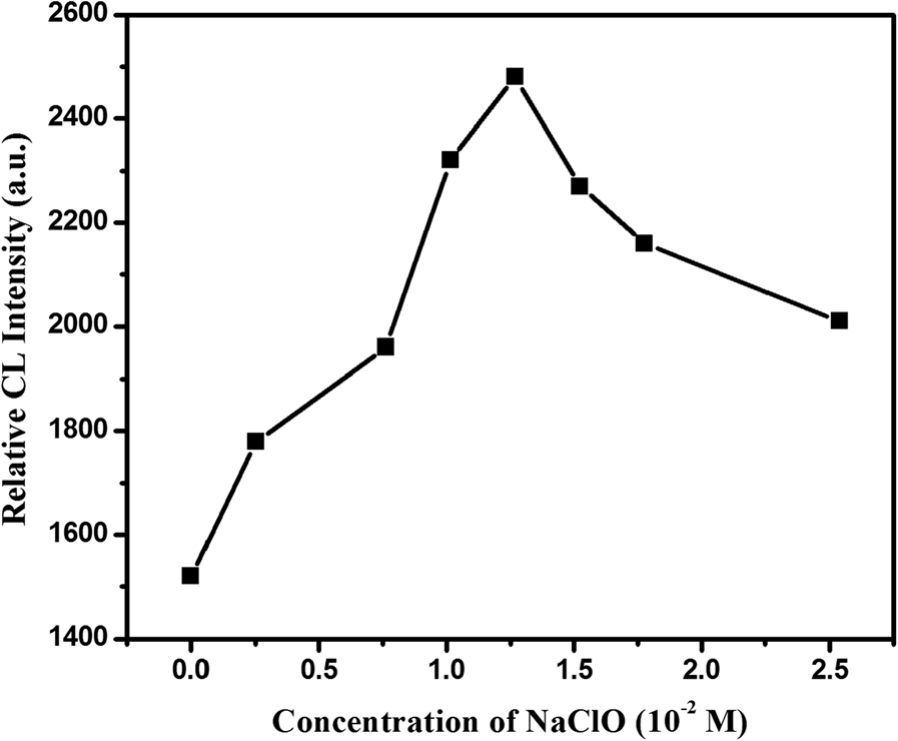
Effect of sodium hypochlorite (NaClO) concentration.

The effect of NaClO concentration on CL emission was investigated in the range of 0 ~ 2.54 × 10^-1^ mol/L (Figure 8), and the CL intensity increased as the NaClO concentration increased from 0 up to 1.27 × 10^-2^ mol/L. However, when the NaClO concentration was more than 1.27 × 10^-2^ mol/L, the CL intensity decreased instead. Therefore, the optimum NaClO concentration, 1.27 × 10^-2^ mol/L, was adopted.

At a lower concentration of NaClO or H_2_O_2_, the signal increases gradually, and the maximum CL intensity occurs at a concentration. Over this concentration, poor relative CL intensity was observed. This may be caused by the increasing of solution viscosity and self-decomposition at high concentration [21,33].

### Effect of pH value

**Figure 9:**
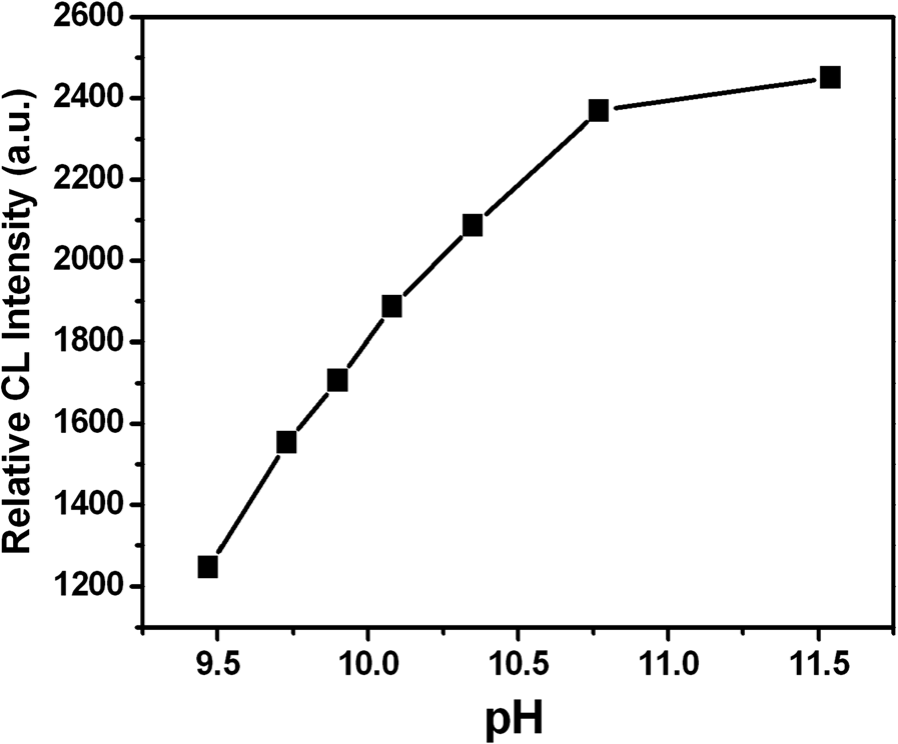
Effect of pH.

It was investigated that the CL signal was stronger under the alkaline condition. The effect of pH buffer solution of NaHCO_3_-Na_2_CO_3_ on CL intensity were investigated in the pH values of 9.47, 9.73, 9.90, 10.08, 10.35, 10.77, and 11.54. The results demonstrated that CL intensity increased with the increase of pH value (Figure 9). The CL intensity achieved its maximum at 11.54. So, NaHCO_3_-Na_2_CO_3_ buffer solution of pH = 11.54 was chosen in the system.

### Determination of estrogens

Under the optimized experimental conditions, the calibration graph of the estrogens showed that the relative CL intensity (*I*) was linearly proportional to the logarithm of the concentration of the estrogen standard solution (*C*). The linear ranges, regression equations, correlation coefficients (*R*), and detection limits obtained were summarized in Table 1. The linear ranges of the determination on estrogens were 3.0 × 10^-6^ ~ 1.0 × 10^-4^ mol/L, 1.0 × 10^-6^ ~ 1.0 × 10^-4^ mol/L, and 1.0 × 10^-6^ ~ 7.0 × 10^-5^ mol/L for estrone, estradiol, and estriol, respectively. And the detection limits were 1.3 × 10^-7^, 3.1 × 10^-7^, and 1.6 × 10^-7^ mol/L for estrone, estradiol, and estriol, respectively.

**Table 1:** **Linear ranges, regression equations, correlation coefficients (*R*), and detection limits of estrogens**

**Estrogen**	**Linear ranges (mol/L)**	**Linear regression equation(*C*** **× 10**^**-7**^ **mol/L)**	***R***	**Detection limit (mol/L)**
Estradiol	1.0 × 10^-6^ ~ 1.0 × 10^-4^	*I* = 4162.13543 - 87.0738C	0.9943	3.1 × 10^-7^
Estriol	1.0 × 10^-6^ ~ 7.0 × 10^-5^	*I* = 3794.98245 - 59.2879C	0.9961	1.6 × 10^-7^
Estrone	3.0 × 10^-6^ ~ 1.0 × 10^-4^	*I* = 3794.20501 - 72.6198C	0.9938	1.3 × 10^-7^

### Selectivity

The selectivity of our approach for detecting estrogen was tested in comparison with some biological species including metal ions, amino acids, and proteins. The concentration of estrogen was 5.0 × 10^-5^ mol/L. The biological species concentration was kept at 0.1 mM. The results were listed in Table 2. The results showed that the system had a good selectivity for estrogen detection.

**Table 2:** Chemiluminescence quenching efficiency in the presence of various biological species

**Species added**	**Chemiluminescence quenching efficiency (%)**
Estradiol	+25.8
Estriol	+20.4
Estrone	+22.4
Na^+^	+0.96
K^+^	+0.73
Ca^2+^	+1.02
Mg^2+^	-0.98
Cu^2+^	+1.13
Zn^2+^	+1.59
Mn^2+^	-0.56
Fe^3+^	+2.03
Glucose	+1.89
BSA	+0.87
Glu	+1.43
IgG	+1.21

### Possible CL reaction mechanism

**Figure 10:**
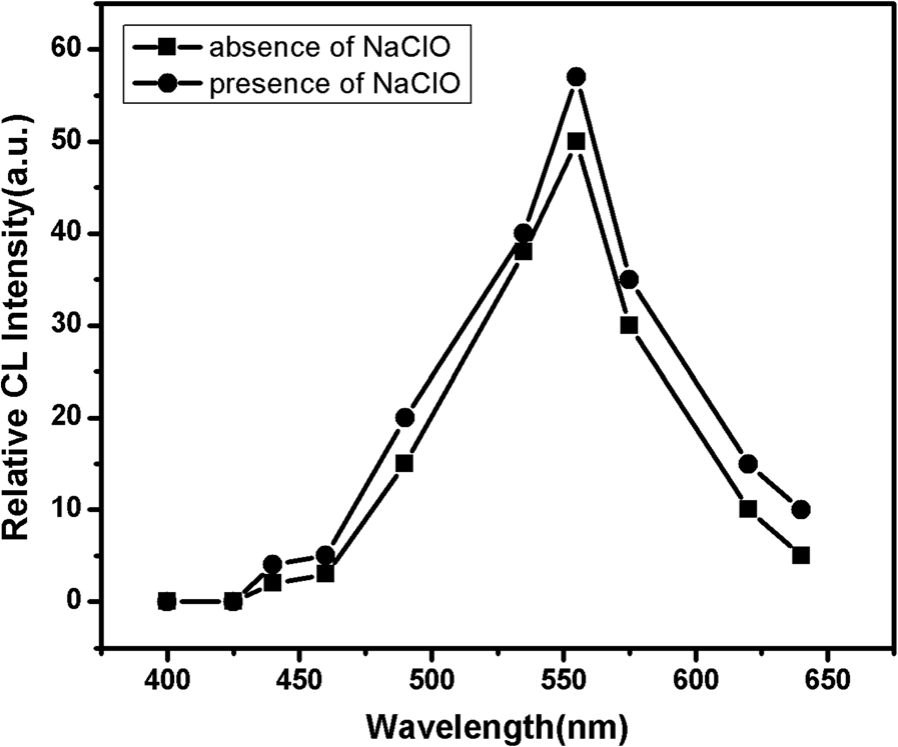
Chemiluminescence spectra of the CL system.

In order to investigate the reaction mechanism of CL enhancement and confirm the emission species, the following experiments were performed. Firstly, the H_2_O_2_-NaClO-CdTe NCs (2.60 nm) CL spectrum was recorded using a BPCL-2-KIC Ultra-Weak Luminescence. The obtained CL spectrum was shown in Figure 8, which clearly indicated that the maximal peak was at 555 nm. As is known, PL spectra of the stable excited states should be identical to CL spectrum, which was demonstrated in our results comparing PL spectra (Figure 3) with CL spectrum (Figure 10). Then, some coexisting substrates (GSH and CdCl_2_ solutions) were injected in turn into H_2_O_2_-NaClO solutions one by one, but no CL signal was found. Therefore, the excited states of the observed CL must be CdTe NCs that were generated in situ during the chemical reaction in the H_2_O_2_-NaClO-CdTe NCs CL system. The states of CdTe NCs, before and after CL reactions, were also examined. It was found that the characteristic peaks of PL emission and UV–Vis absorption for CdTe NCs disappeared after CL reactions. These results demonstrated that the nanocrystal lattice structure of CdTe NCs has been destroyed completely after being oxidized by enough H_2_O_2_. Thus, the CL reaction can be described in its simplest form as follows:

(1) $$\text{Oxidant}+\text{CdTe}\;\text{NCs}\to {\left(\text{CdTe}\;\text{NCs}\right)}^{*}+h\nu$$

where (CdTe NCs)* refers to the excited state of CdTe NCs.

Therefore, the possible mechanism of the enhanced CL reaction induced by CdTe NCs can be concluded with a simple form as shown below:

(2) $$\text{NaClO}+H_{2}O\to \text{HClO}+\text{NaOH}$$

(3) $$2\text{HClO}\to 2\text{HCl}+O_{2}$$

(4) $$\text{GSH}+O_{2}+{\text{OH}}^{-}\to O_{2}^{-}+\text{RS}+H_{2}O$$

(5) $$O_{2}^{-}+\text{CdTe}\to \text{CdTe}({e^{-}}_{1\mathrm{Se}})+O_{2}$$

(6) $$O_{2}^{-}+H_{2}O_{2}\to {\text{OH}}^{•}+{}^{1}O_{2}$$

(7) $${\text{OH}}^{•}+\text{CdTe}\to {\text{OH}}^{-}+\text{CdTe}({h^{+}}_{1\text{Sh}})$$

(8) $${{\text{CdTe}(h}^{+}}_{1\text{Sh}})+\text{CdTe}({e^{-}}_{1\text{Se}})\to {\left(\text{CdTe}\right)}^{*}\to \text{hv}$$

## Conclusion

A flow-injection CL method has been established for determination on estrone, estradiol, and estriol based on the inhibition of CdTe-hydrogen peroxide CL system enhanced by sodium hypochlorite. The method has the merits of high sensitivity, and wide linear ranges. It is a new principle and alternative method for detection on estrogens and extends the analytical application of CdTe CL system.

## Competing interests

The authors declare that they have no competing interests.

## Authors’ contributions

BL, JB, and HD carried out the experimental work, participated in the planning of the experiment and drafted the manuscript. ZP and LD participated in the argument on this manuscript and the manuscript was touched up by them. All authors read and approved the final manuscript.
